# The efficacy of disinfectants in the decontamination of dental unit water lines: an *in vitro* laboratory study

**DOI:** 10.1038/bdjopen.2016.3

**Published:** 2016-02-26

**Authors:** Mrudula Patel, Jainisha Desai, Peter C Owen

**Affiliations:** 1 Division of Oral Microbiology, Department of Oral Biological Sciences, School of Oral Health Sciences, Faculty of Health Sciences, University of The Witwatersrand, Johannesburg, South Africa; 2 Department of Oral Rehabilitation, School of Oral Health Sciences, Faculty of Health Sciences, University of The Witwatersrand, Johannesburg, South Africa

## Abstract

**Objectives/Aims:**

This *in vitro* laboratory study compared the efficacy of water, sodium percarbonate (SPC) and chlorine dioxide (ClO_2_) solutions in the disinfection of dental unit water lines (DUWLs).

**Materials and Methods:**

New DUWL tubes were cut, split open, and mono-culture and mixed-culture biofilms of *Staphylococcus aureus*, *Enterococcus faecalis* and *Streptococcus mutans* were grown. Harvested biofilms from the sectioned DUWL tubes were exposed to sterile distilled water, SPC or 5 and 10 p.p.m. ClO_2_ in both a stationary phase and through a constant flow. Bacterial counts were compared using the Kruskal–Wallis nonparametric rank test.

**Results:**

In the mono-culture biofilms, SPC, 5 and 10 p.p.m. ClO_2_ significantly reduced all the test organisms (*P*<0.01). However, no significant difference was found between SPC and ClO_2_. In the mixed-culture biofilms exposed to disinfectant without flow, ClO_2_ significantly reduced the biofilm (*P*=0.02) compared with water and SPC. Similarly, in the constant flow study, ClO_2_ proved to be superior to water.

**Conclusion:**

At low concentrations, ClO_2_ with and without flow significantly reduced the mixed-culture biofilm grown *in vitro* on the sections of the DUWL tubes. Therefore, it has the potential to be used in the patient treatment water, as it is potable at these concentrations, and to decontaminate and limit the biofilm formation in the water lines.

## INTRODUCTION

The dental chair unit contains water lines that supply water from a reservoir or municipal supply to the handpieces, the triplex air/water syringe and spittoon. These lines vary in thickness and material construction, and become contaminated with environmental microorganisms, pathogens and opportunistic pathogens. These bacteria create biofilms on the tube walls that provide nutrients and protection to one another.^1^ The flow of water in the dental unit water lines (DUWLs) generates shear forces that detach pieces of biofilm and planktonic forms of microorganisms and their endotoxins. Contamination of DUWLs therefore becomes a potential source of infection particularly for immunocompromised patients and is hazardous for both patients and dental health-care personnel. The source of contamination is tap water or retrograde aspiration of oral secretions by handpieces.^2,3^

Among the many physical methods that can improve the microbiological quality of DUWL output water, the use of disinfectant is the most efficacious means of ensuring decontamination. Chemicals such as sodium percarbonate (SPC), hydrogen peroxide and sodium perborate have been tested and they have shown variable efficacy^4^ mainly owing to the different test concentrations and the type of interventions. Most studies have tested the efficacy of disinfectants as flushing solutions with intermediate use of a normal water supply. However, disinfectants may only be effective when the development of biofilm is minimised and the intermittent exposure of normal water is also eliminated. Disinfectants used in DUWLs have to not only eliminate heterotrophic bacteria but also common pathogens. In addition, the disinfectant has to be safe and biocompatible. This study compared the efficacy of a commonly used product, SPC and newly available product, chlorine dioxide (ClO_2_), in the disinfection of DUWLs and their possible use during patient treatment.

## MATERIALS AND METHODS

### Cultures and inoculums

*Staphylococcus aureus* ATCC 2943, *Enterococcus faecalis* ATCC29121 and a clinical strain of *Streptococcus mutans* were used in the study. Ethical clearance was obtained from the Human Research Ethics committee of The University of Witwatersrand, Johannesburg. Cultures were grown on blood agar plates, and inoculums were prepared to obtain an optical density of 0.5 at 405 nm (~10^6^–10^7^ c.f.u./ml).

### Test products

These products are commercially available and the solutions were prepared according to the manufacturer’s instructions. The SPC tablet (SPC, *N*-alkyl dimethyl benzyl ammonium chloride, *N*-alkyl dimethyl ethylbenzyl ammonium chloride and silver nitrate) is known as A-dec ICX (A-dec, Newberg, OR, USA), and 5 and 10 p.p.m. of slow release ClO_2_ tablets known as SteriWright (Wright Millners, Johannesburg, South Africa). Sterile distilled water was used as a control to represent flushing.

### Standardisation of technique

Three-centimetre segments of standard-sized yellow (water supply to spittoon), white (to auxiliary devices) and grey (to the air turbines) new DUWL tubes (A-dec) were cut and slit into two halves. The tubes were decontaminated with 70% alcohol and by placing them under ultraviolet light for 48 h. To grow biofilms, the tubes were placed in Brain Heart Infusion broth, inoculated with 100 μl of the appropriate culture inoculum and incubated at 37 °C for 72 h. They were rinsed with sterile distilled water to remove residues of media and unattached bacterial cells, and the biofilms were then scraped off with swabs. The swabs and the tubes were placed in 5 ml sterile distilled water, vortexed, and bacterial counts were obtained on blood agar plates using the serial dilution technique. These experiments were repeated five times, and the results were analysed using a one-sample *t*-test.

### Efficacy of disinfectants—*in vitro* study

For the mono-culture experiments, biofilms were grown on five sets of the yellow, white and grey tubes. One set of tubes with biofilms were cultured for the quantity of bacteria using the serial dilution technique (control count). The other four sets were allowed to stand in either of the test chemicals or water for 24 h. They were then removed, and the standardised procedure described above was followed to obtain the bacterial counts.

For the mixed-culture experiments, biofilms were grown using all three cultures of bacteria. In this case, the bacterial counts were obtained using Baird–Parker agar (*S. aureus*), MacConkey agar (*E. faecalis*) and Mutans Bacitracin agar plates (*S. mutans*).

For both the experiments, the percentage kill was calculated using control counts of unexposed tubes using the formula:

(controlcounts−testcounts/controlcounts)×100=percentagekill.

All the experiments were repeated three times. These data were compared using Kruskal–Wallis nonparametric rank tests.

### Efficacy of disinfectants—simulation study

A fish-tank pump was used to simulate the dental unit. This pump had the capacity to pump 400 litres of water per hour. This experiment was repeated using only the grey tubes. A 25-cm segment of tube was cut, fitted onto a pump and submerged into a beaker containing 70% alcohol. The pump was switched on for 2 h to decontaminate the pump and tube. The tube was removed and placed under ultraviolet light. After 24 h, the pump was removed from the alcohol, drained thoroughly, air dried and the decontaminated tube was reconnected. This assembled system was submerged into 650 ml of Brain Heart Infusion broth. The media were inoculated with 1 ml of mixed-culture inoculum, incubated for 72 h with a change of medium every day. The pump was switched on for 8 h a day and the rest of the time the tubes were left in the same medium. A 3-cm segment was cut, wiped with 70% alcohol and split open using a sterile scalpel. Biofilms from the inner walls of the tube were then scraped off with swabs. The swabs and the tubes were placed in 5 ml sterile distilled water, vortexed and bacterial counts were obtained using Baird–Parker agar (*S. aureus*), MacConkey agar (*E. faecalis*) and Mutans Bacitracin agar plates (*S. mutans*). The bacterial counts were taken as control counts. The rest of the tubing was also wiped with 70% alcohol, reconnected to a decontaminated pump, submerged into sterile distilled water, switched on for 8 h and kept in the same sterile distilled water at room temperature for 24 h. Five 3-cm segments were cut, the outer surface was decontaminated and the biofilm from the inner wall was cultured. Similarly, 72-h biofilms were exposed to SPC, and 5 and 10 p.p.m. ClO_2_ solutions. These experiments were repeated five times for each disinfectant. The percentage kill data for each organism were pooled to provide 15 readings per disinfectant, which were compared using Kruskal–Wallis nonparametric rank tests.

## Results

### Standardisation of technique

The mean biofilm counts of *S. aureus*, *E. faecalis* and *S. mutans* was 1.3×10^7^, 2.6×10^7^ and 1.0×10^5^ per tube. No significant difference in the bacterial counts between the repeats per tubes and between all the tubes was found (*S. aureus*: *P*=0.06, *E. faecalis*: *P*=0.15, *S. mutans*: *P*=0.5). Therefore, it was determined that the technique was standardised. However, the adherence ability of *S. mutans* was found to be weaker than *S. aureus* and *E. faecalis*. In addition, the adherence ability of the test organisms was not tube dependent.

When the efficacy of the disinfectants was compared between the tubes no significant difference in the % kill was found (*P*=0.6). When the efficacy of the disinfectants was compared between the test organisms, no significant difference in the % kill was found (*P*=0.4). Therefore, all the results were combined (% kill for the all the tubes and all the organisms, *n*=27) and the efficacy of disinfectants were compared.

### Efficacy of disinfectants

**Figure 1:**
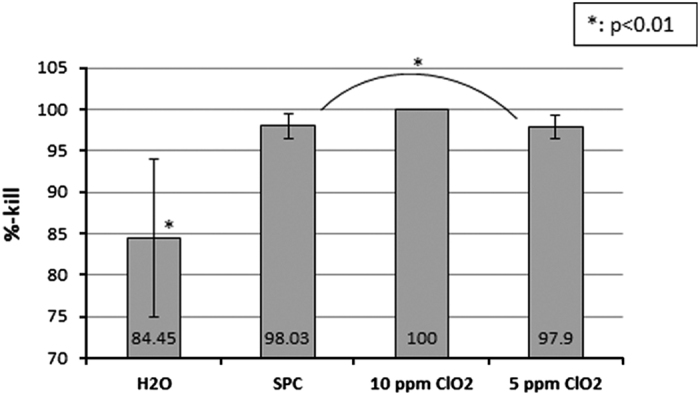
Comparison of disinfectants in the disinfection of yellow, white and grey tubes contaminated individually (mono-culture) with *S. aureus, E. faecalis* and *S. mutans*. where *n*=27.

The bacterial counts are shown in the Table 1, which shows the mean number of challenged organisms and the reduction in the number of organisms. Continuous pumping of ClO_2_ showed complete removal of mixed-culture biofilm. For each test, these bacterial counts were converted into % kill. When the disinfectants were compared using % kill, the results (Figure 1) showed that in the mono-culture biofilm experiments, SPC, 5 p.p.m. ClO_2_ and 10 p.p.m. ClO_2_ had significantly high (*P*<0.01) % kill compared with water. However, there was no difference in the % kill between the test disinfectants.

**Figure 2:**
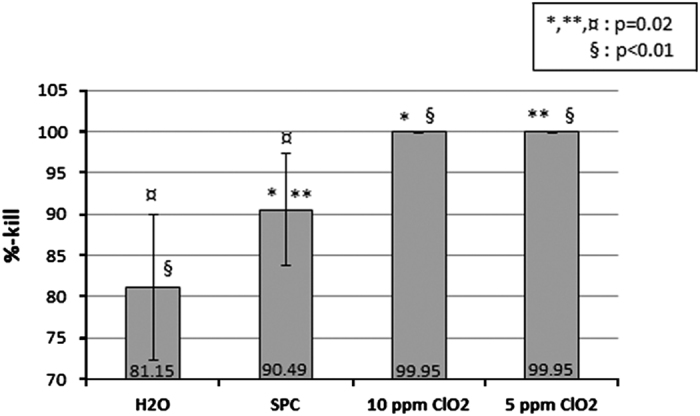
Comparison of disinfectants in the disinfection of yellow, white and grey tubes contaminated with mixed-cultures of *S. aureus, E. faecalis* and *S. mutans* in an *in vitro* stationary environment. Bacterial counts for each organism were pooled. where *n*=27.

In the mixed-culture biofilm experiments, the test disinfectants had significantly higher % kill compared with water (Figure 2). Both concentrations of ClO_2_ also had significantly higher % kill compared with SPC (*P*=0.02) and no difference in the % kill of the two concentrations of ClO_2_.

**Figure 3:**
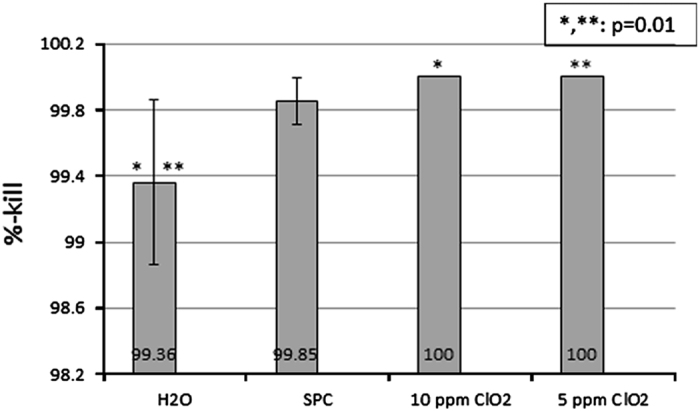
Comparison of disinfectants in the disinfection of simulation pump-attached grey tubes contaminated with mixed-cultures of *S. aureus, E. faecalis* and *S. mutans*. Bacterial counts for each organism were pooled. where *n*=15.

In the mixed-culture biofilm pump experiments (Figure 3), SPC did not prove any different from water (*P*=0.1). However, both the concentrations of ClO_2_ proved to be better than water (*P*=0.01).

## Discussion

Although data related to transmission of infections due to DUWL are under reported, it is still a threat especially to immunocompromised patients.^4^ This study has shown that the test chemicals can significantly reduce the biofilms compared with water, which suggest that flushing the unit with water alone will not be adequate.^3,5,6^ Many products containing disinfectants are available and they have been shown to have variable efficacy purely because of the unique nature of the DUWL system.^4^ It becomes contaminated with different types of bacteria, which forms highly structured microbial communities that are difficult to remove.

This study has shown that biofilm cotaining mixed flora could not be eliminated with SPC (Table 1). Nevertheless, SPC performed slightly better when the test organisms were challanged individually and used in the pump system. It has been suggested that sodium perborate and sodium carbonate, which are similar to SPC, generally have variable efficacy.^4^ Similarly, the test product SPC that contained SPC, surfactants with biocidal activities and antimicrobial compound silver nitrate also displayed variable efficacy. In a study, sodium carbonate together with sodium carbonate peroxy hydrate has shown inconsistant results at different time intervals.^6^ It has been reported that SPC can efficiently eliminate heterotrophic bacteria and produce water containing bacterial counts within the permitted quantity of <500 c.f.u./ml^7–9^ and yet we failed to show this phenomenon. Although the efficacy of all the disinfectants improved in the pump study compared with the stationary experiments, SPC did not prove to be better than water. On the other hand, ClO_2_ consistently showed better results than water.

As the results in our study have shown, Environmental Protection Agency (EPA) approved ClO_2_ is an excellent biocide and its use in DUWL has been recommended.^1,6^ However, Smith *et al.* ^10^ reported that up to 50 p.p.m. ClO_2_ did not consistently provide potable quality water from an intermittently treated DUWL, although it reduced the total viable bacteria. In contrast, 3 p.p.m. ClO_2_ was reported to produce water with <500 c.f.u./ml of heterotrophic bacterial counts.^11^ Similarly, the use of ClO_2_ (unknown concentration) in DUWLs was shown to maintain <10 c.f.u./ml of bacteria for up to 11 days.^6^ This suggests that to achieve a significant beneficial effect, ClO_2_ should be used continuously and not intermittently.^5,6,12^ Continuous use of ClO_2_ in the DUWLs may prevent biofilm formation by reducing the number of bacteria that will also improve the quality of water.

Regular use of disinfectant may also create other problems particularly with chlorinated compounds as they are considered corrosive. ClO_2_ is reported to cause no corrosion at neutral pH, but can cause corrosion at an acidic pH of 2–3 and at very high concentrations.^13^ It is extremely unlikely that such an acidic pH would ever occur in DUWLs, which suggests that corrosion may not be a problem. It has been shown that the maintainance of the DUWL system can be achieved either by flushing with a disinfectant regularly or by adding a low concentration of disinfectant to the treatment water. However, the low concentrations of SPC studied here were ineffective in reducing the bacterial counts to <100 and even <200 c.f.u./ml as recommended by European Union and American Dental Association, respetively.^14,15^ This study has shown that continuous use of 5 p.p.m. concentration of ClO_2_ was effective in killing pathogenic bacteria present in the form of biofilm on the DUWLs and this concentration is acceptable in the treatment water. EPA and Centers for Disease Control and Prevention (CDC) allow 8 p.p.m. ClO_2_ in drinking water.^16^ Therefore, 5 p.p.m. ClO_2_ may be safely used to continuously disinfect the DUWL tubes and water during dental operations. Addition of disinfectant into the treatment water is easy and less time-consuming.

One of the limitations of this study is that actual dental chair units were not used. However, an artificial pump system was created to simulate the DUWL system, as also shown by Spratt *et al.* ^17^ In this simulated system, mixed-culture biofilms of three common pathogens were grown and decontaminated successfully, validating the results. However, further research is required to test this product in the DUWLs to establish its *in situ* and long-term efficacy. One of the limitations of *in situ* studies, however, is that the output water is usually tested, which contains only a fraction of bacteria detached from the biofilm. The number of challenged bacteria is important, and in *in vitro* studies at least known quantities of bacteria including pathogens can be challenged, which is perhaps more representative of the clinical setting.

## Conclusion

This study has shown that 5 p.p.m. ClO_2_ can kill 99.95% of mixed-culture biofilm grown *in vitro* on the sections of the DUWL tubing and it is potable at this concentration, therefore has the potential to be used as a disinfectant in these lines and to be used in the treatment water. Further research is required to assess the DUWL disinfectant efficacy of ClO_2_ in a normal clinical setting and to ascertain whether it might result in adverse corrosion of dental surgery equipment.

## Figures and Tables

**Table 1:** Effect of water, SPC and ClO_2_ on the biofilms of *Staphylococcus aureus, Enterococcus faecalis* and *Streptococcus mutans* on the dental unit waterline yellow, white and grey tubing

*Experiments*	*Mean±s.d. (bacterial counts in c.f.u./ml)*				
	*Control*	*H*_*2*_ *O*	*SPC*	*5 p.p.m. ClO*_*2*_	*10 p.p.m. ClO*_*2*_
Stationary^a^ (*n*=27)	8.3×10^6^±1.8×10^7^	3.4×10^5^±8.8×10^5^	5.1×10^4^±8.5×10^4^	7±38	0±0
Stationary^b^ (*n*=27)	7.0×10^5^±1.7×10^6^	6.8×10^4^±2.3×10^5^	1.8×10^4^±3.6×10^4^	1.9×10^2^±5.6×10^2^	65±1.5×10^2^
Pump study^c^ (*n*=15)	5.7×10^5^±6.6×10^5^	4.2×10^3^±6.4×10^3^	4.5×10^2^±7.6×10^2^	0±0	0±0

Abbreviations: ClO_2_, chlorine dioxide; SPC, sodium percarbonate.

a Mono-culture biofilm on each type of tubing repeated three times.

b Mixed-culture biofilm on each type of tubing repeated three times (bacterial counts obtained for each organism).

c Mixed-culture biofilm on the grey tubing only repeated five times (bacterial counts obtained for each organism).
